# Central serous chorioretinopathy in primary hyperaldosteronism

**DOI:** 10.1007/s00417-016-3417-8

**Published:** 2016-07-08

**Authors:** Elon H. C. van Dijk, Michiel F. Nijhoff, Eiko K. de Jong, Onno C. Meijer, Aiko P. J. de Vries, Camiel J. F. Boon

**Affiliations:** 1 Department of Ophthalmology, Leiden University Medical Center, Department J3-S, PO Box 9600, 2300RC Leiden, Netherlands; 2 Department of Medicine, Division of Nephrology and Transplantation, Leiden University Medical Center, Leiden, Netherlands; 3 Department of Medicine, Division of Endocrinology and Metabolism, Leiden University Medical Center, Leiden, Netherlands; 4 Department of Ophthalmology, Radboud University Medical Center, Nijmegen, Netherlands; 5 Einthoven Laboratory for Experimental Vascular Medicine, Leiden University Medical Center, Leiden, Netherlands

**Keywords:** Central serous chorioretinopathy, Eplerenone, Hyperaldosteronism, Mineralocorticoid antagonist, Cross-sectional study, Spironolactone

## Abstract

**Purpose:**

To describe ophthalmological characteristics of 13 patients with primary hyperaldosteronism (PA).

**Methods:**

Cross-sectional study. All patients underwent extensive ophthalmological examination.

**Results:**

Thirteen PA patients (9 male, 4 female) were diagnosed with arterial hypertension for 11.0 ± 11.2 years. Ophthalmological imaging revealed macular serous subretinal fluid (SRF) on optical coherence tomography in 2 patients (15 %). In one of these patients, bilateral chronic central serous chorioretinopathy (CSC) with polypoidal choroidal neovasculopathy was diagnosed, which was effectively treated with full-dose photodynamic therapy. In the other patient with SRF and bilateral diffuse hyperfluorescent areas on fluorescein angiography, the SRF had decreased spontaneously after 6 weeks of follow-up. In 5 of the remaining patients (38 %), retinal pigment epithelium alterations resembling findings characteristic for CSC were seen on multimodal imaging. The mean subfoveal choroidal thickness was 290.2 ± 65.0 μm.

**Conclusions:**

Retinal abnormalities resembling (subclinical) CSC are common in patients with PA. These findings indicate that mineralocorticoid-mediated pathways are involved in the pathogenesis of CSC. In CSC patients with hypertension of unknown origin, a diagnosis of PA should be considered.

## Introduction

Central serous chorioretinopathy (CSC) is a chorioretinal disease, characterized by a neuroretinal detachment due to the presence of serous subretinal fluid (SRF), as a result of dysfunction of the retinal pigment epithelium (RPE), and hyperpermeability and thickening of the underlying choroid. The exact pathogenetic mechanism of CSC still has to be unravelled [1–5]. CSC is more common in men, and presents with complaints such as vision loss, image distortion, and loss of color and contrast vision [2–4, 6, 7]. Genetic risk factors may play a role in CSC, possibly via the complement system [8–10]. Exposure to both endogenous (endogenous hypercortisolism/Cushing’s syndrome) and exogenous corticoids is strongly associated with CSC [11–14]. However, the pathogenetic explanation for the link between CSC and corticosteroids is currently unclear. In the pathogenesis of CSC, both the glucocorticoid receptor (GR) and the mineralocorticoid receptor (MR), a receptor with equal affinity for glucocorticoids and for mineralocorticoids such as aldosterone, have been postulated to play a role [2].

In a rat model, overstimulation of the MR resulted in choroidal changes resembling findings in CSC patients [15]. Several pilot studies have indicated that there may be a therapeutic role for the MR antagonists spironolactone and eplerenone in the treatment of CSC [16–19]. However, the effect of these antagonists on central macular and choroidal thickness (CT) and on complete and durable resolution of SRF after cessation of therapy is unclear, and randomized controlled trials are currently lacking [18–20]. Although the increased incidence of CSC in patients with endogenous hypercortisolism is well-known [14, 21, 22], no studies have been published on the occurrence of CSC in patients with primary aldosteronism (PA, primary hyperaldosteronism) thus far.

PA is characterized by the overproduction of aldosterone by one or both of the adrenal glands, independent of its normal regulator angiotensin II. Unilateral adrenal adenoma (Conn’s disease) and bilateral adrenal hyperplasia account for more than 90 % of PA cases. For these diseases laparoscopic adrenalectomy and MR antagonists such as spironolactone and eplerenone are recommended, respectively. PA due to both adrenal and non-adrenal aldosterone-producing tumors, primary unilateral adrenal hyperplasia, and familial hyperaldosteronism occurs less frequently [23].

Although the association between endogenous hypercortisolism and CSC is well-established [14, 21, 22], and there may be a role for MR and treatment with MR antagonists in CSC, it is unknown if PA is also associated with CSC-like abnormalities. Therefore, in this study we examined patients with PA to detect clinical findings indicative of (subclinical) CSC.

## Methods

### Patient selection

In this cross-sectional study, 13 patients with PA were referred to the Department of Ophthalmology of the Leiden University Medical Center for ophthalmological analysis. PA patients in whom diagnosis had been established between May 2008 and August 2015 were asked to take part in this study. Both patients who already received either surgery or medication because of the hyperaldosteronism and patients who had not received any PA treatment were included in this study. One patient with a pituitary adenoma, influencing the hypothalamic-pituitary-adrenal axis, was excluded because of a known relationship of this disease with CSC [14, 21, 22]. Written informed consent for the enrollment was obtained from all subjects. The study adhered to the tenets of the Declaration of Helsinki. Approval of the institutional review board and the ethics committee was obtained (NL50816.058.14).

### Ophthalmological imaging

All patients received complete ophthalmic examination, including Early Treatment of Diabetic Retinopathy Study (ETDRS) best-corrected visual acuity (BCVA) measurement. After this measurement, pupils were dilated by using 1 % tropicamide and 5 % phenylephrine. Indirect ophthalmoscopy and digital color fundus photography (Topcon Corp., Tokyo, Japan) were performed, and optical coherence tomography (OCT), enhanced depth imaging (EDI-)OCT, fundus autofluorescence, and oral fluorescein angiography (FA) images were obtained, using spectral-domain OCT (Spectralis HRA + OCT; Heidelberg Engineering, Dublin, CA, USA). EDI-OCT was used to measure subfoveal CT. Images were evaluated by an experienced retina specialist (CJFB). Oral FA was performed after 10 milliliters of 20 % fluorescein was administered orally after a fasting period of at least 3 hours, and photos were taken at 10, 15, 20, 25, and 30 minutes after administration. When ophthalmological imaging revealed findings requiring treatment and/or follow-up based on the judgment of the treating ophthalmologist, these visits were scheduled at our outpatient clinic. Based on the results of the ophthalmological screening, indocyanine green angiography using the spectral-domain OCT could also be performed if deemed necessary.

## Results

### Patient characteristics

The mean age of the 13 PA patients (9 male, 4 female) was 55.1 ± 12.0 years (range, 36–74 years). All patients had arterial hypertension, which is the most prominent clinical sign of PA [23]. Hypertension was diagnosed 11.0 ± 11.2 years (range, 0.5–43 years) before ophthalmological evaluation. No significant difference between the patients with and without ophthalmological abnormalities could be detected regarding both patient age and duration of pre-existing hypertension. At the moment of PA presentation, hypertension was treatment-resistant (need of prescription of >2 antihypertensive agents) in 8 patients (62 %). At the time of ophthalmological phenotyping, two patients still had hypertension despite antihypertensive treatment. Hypokalemia, a sign of relatively severe PA [24], developed either spontaneously or after the use of diuretics and always in combination with hypertension [23], was a presenting symptom of PA in 10 patients (77 %). Obstructive sleep apnea syndrome was previously diagnosed in one patient. No other diseases known to be possibly associated with CSC were present in the included patients.

At the time of the visit to the Department of Ophthalmology, 3 patients were on chronic spironolactone treatment and 3 patients used eplerenone as antihypertensive treatment, previously prescribed by the endocrinologist for a mean duration of 2.2 years (range, 10 weeks – 7 years). One patient reported the current local nasal daily use of the steroid fluticasone, because of allergic rhinitis. Two other patients reported a previous short course of oral steroid treatment in 2012 and cutaneous steroid treatment in 2013, respectively. No patients reported the use of either sildenafil or tadalafil.

PA due to unilateral adrenal adenoma was diagnosed in seven patients; bilateral adrenal hyperplasia was detected in three patients. In one patient, the cause of PA was not found. Six patients had undergone unilateral laparoscopic adrenalectomy at the time of the ophthalmological evaluation. Another patient was already scheduled for surgery and was prescribed eplerenone and nifedipine at the moment of ophthalmological analysis. After surgery, pathological analysis revealed hyperplasia of the excised adrenal gland. The last patient was scheduled to receive adrenal vein sampling to detect the origin of PA. Eleven out of the other 12 patients were treated with antihypertensive drugs, including the three patients who used spironolactone and three patients who received eplerenone for this purpose. Clinical characteristics of the patients are summarized in Table 1.

**Table 1:** Clinical characteristics of patients with primary hyperaldosteronism (PA)

Patient	Age	Gender	Type (Type of Surgery)	Systemic Presentation of PA	Current Medication
1	72	M	Adrenal adenoma (−)	Hypertension	Amlodipine, enalapril, phenprocoumon, propafenone, spironolactone
2	64	F	Unknown origin (−)	Therapy-resistant hypertension	Colecalciferol/calcium carbonate, levothyroxine, metoprolol, spironolactone
3	59	M	Adrenal adenoma (laparoscopic adrenalectomy)	Therapy-resistant hypertension, hypokalemia	Colecalciferol, doxasozin, furosemide, nifedipine, omeprazole
4	62	F	Adrenal hyperplasia (−)	Hypertension, hypokalemia	Amlodipine, colecalciferol/calcium carbonate, melatonin, simvastatin, potassium chloride, spironolactone
5	42	M	Adrenal adenoma (laparoscopic adrenalectomy)	Therapy-resistant hypertension, hypokalemia	Amlodipine, calcium carbonate, metoprolol, perindopril
6	41	M	Adrenal adenoma (laparoscopic adrenalectomy)	Hypertension, hypokalemia	Nifedipine
7	50	M	Adrenal adenoma (laparoscopic adrenalectomy)	Therapy-resistant hypertension, hypokalemia	Barnidipine
8	36	F	Adrenal adenoma (laparoscopic adrenalectomy)	Hypertension, hypokalemia	–
9	58	M	Adrenal hyperplasia (laparoscopic adrenalectomy)	Therapy-resistant hypertension, hypokalemia	Amlodipine, enalapril
10	74	M	Adrenal adenoma (−)	Therapy-resistant hypertension, hypokalemia	Amlodipine, eplerenone, losartan
11	48	M	Adrenal hyperplasia (laparoscopic adrenalectomy)	Hypertension, hypokalemia	Eplerenone, metformin, nifedipine
12	58	M	Adrenal hyperplasia (−)	Therapy-resistant hypertension, hypokalemia	Acetylsalicylic acid, barnidipine, diclofenac, eplerenone, fluticasone, perindopril, potassium chloride
13	45	F	Unknown origin (scheduled for adrenal vein sampling)	Therapy-resistant hypertension	Amlodipine, hydrochlorothiazide

### Ophthalmic characteristics

Ophthalmologic history taking revealed one 64-year-old female patient who reported unilateral blurred vision and metamorphopsia. This patient had been visiting another hospital since 2012 because of ‘early age-related macular degeneration’. Ophthalmological history revealed that two other patients had been previously diagnosed with venous hemi-occlusion, for which scatter laser coagulation treatment was performed. Another patient was previously diagnosed with a choroidal naevus. Ophthalmic family history was unremarkable except for one PA patient who reported a mother who was previously diagnosed elsewhere with age-related macular degeneration. Mean ETDRS BCVA of the 26 eyes was 89.0 ± 6.5 letters (range, 67–99 letters), with a mean spherical equivalent of the manifest refraction of 0.19 ± 2.39 diopters (range, −6.25 to +5 diopters).

**Fig. 1:**
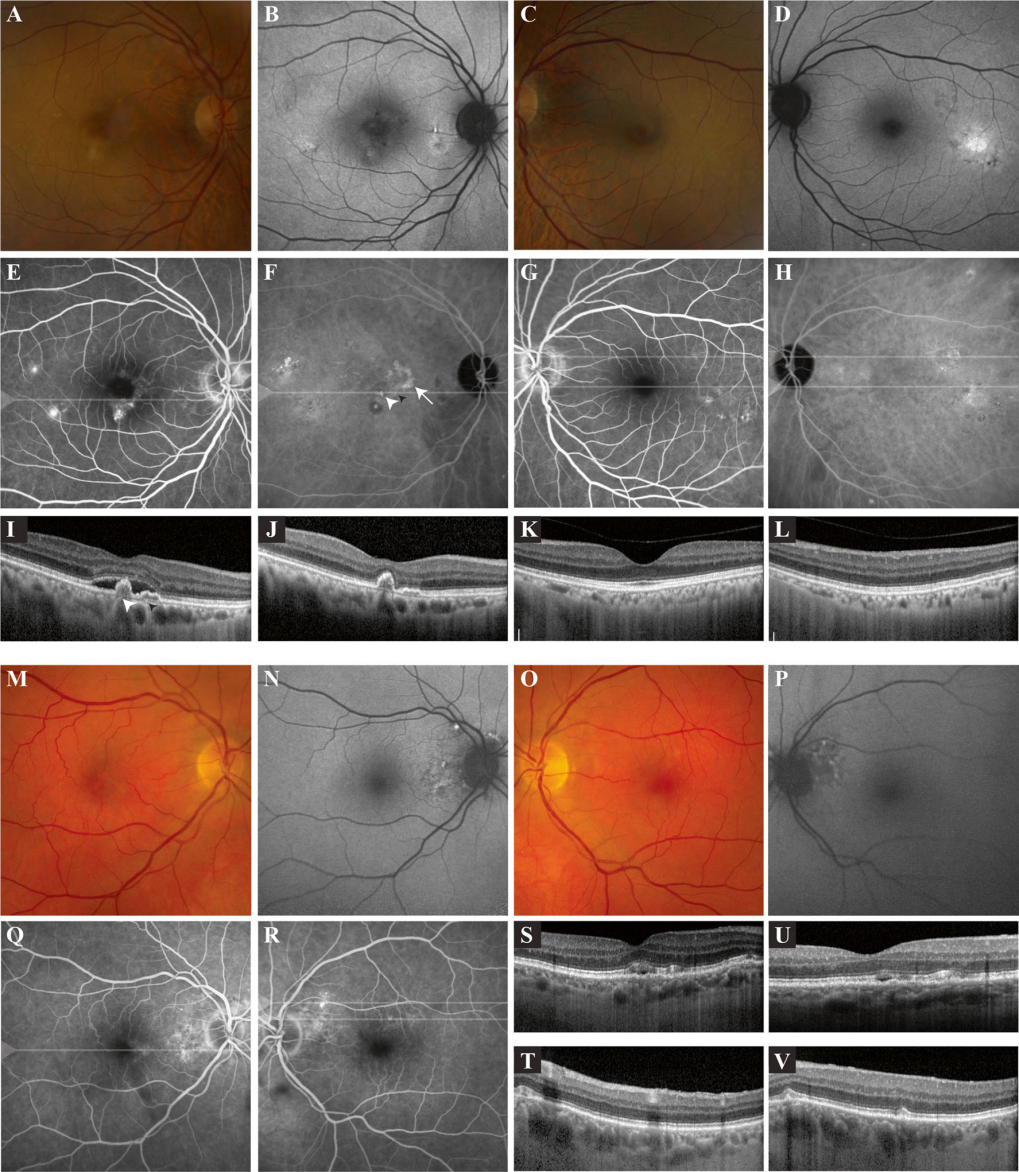
Ophthalmological imaging of two patients with primary hyperaldosteronism in whom foveal subretinal fluid (SRF) was detected at screening. **a–l**. Multimodal imaging of a 64-year-old female with primary hyperaldosteronism. Fundus photography of the right eye (**a**) revealed central irregular yellowish changes in the fovea and milder focal pigmentary changes in the temporal macula, with multifocal autofluorescence changes on fundus autofluorescence (FAF; **b**). Fundus photography of the left eye (**c**) also showed mild retinal pigment epithelium (RPE) abnormalities, with both hypo- and hyperautofluorescent changes on FAF temporally in the macula (**d**). On fluorescein angiography (FA) of the right eye (**e**) two hot spots of leakage were present in the temporal macula, as well as a hyperfluorescent spot inferior to the fovea. Indocyanine green angiography (ICGA) revealed a lesion suspicious for polypoidal choroidal vasculopathy in the right eye (*white arrowhead*), possibly on the border of an area of an occult neovascularisation (*white arrow*; **f**). Hyperfluorescent areas compatible with chronic central serous chorioretinopathy (CSC) on ICGA of the left eye (**h**) were larger as compared to the areas on FA (**g**). Optical coherence tomography (OCT) revealed foveal SRF in the right eye (**i**), with the presence of a round, dome-shaped RPE detachment (*large white arrowhead* in *i*) corresponding to the small round polyp on ICGA (*white arrowhead* in *f*), in association with a more shallow RPE detachment (“double layer sign”, *small black arrowhead* in *i*) that may correspond to the possible occult neovascularisation seen on ICGA (*small black arrowhead* in f). Moreover, extrafoveal RPE alterations were found on OCT. At baseline, subfoveal choroidal thickness (CT) was 235 μm in the right and 206 μm in the left eye. The patient underwent full-dose photodynamic therapy in the right eye, refusing anti-vascular endothelial growth factor treatment as an additive treatment. At the evaluation visit 6 weeks after therapy, the SRF in the right eye (**j**) had disappeared, with the RPE detachments still present. No foveal SRF was present on OCT in the left eye (*k*; lower scan in *g* and *h*). An extrafoveal OCT of this eye (*l*; upper scan in *g* and *h*) revealed RPE changes temporally in the macular region. At the evaluation visit, subfoveal CT in the right eye had decreased to 220 μm. **m–v**. Multimodal imaging of a 74-year-old male patient with primary hyperaldosteronism. Fundus photography of the right eye (**m**) showed mild foveal and extrafoveal RPE abnormalities, and mild irregular peripapillary changes on FAF (**n**). Fundus photography of the left eye (**o**) revealed mainly extrafoveal RPE changes, with the same picture on FAF (**p**) as in the right eye. FA of the right (**q**) and left eye (**r**) showed multifocal hyperfluorescent areas. The foveal OCT scan of the right eye (**s**) showed shallow serous SRF and RPE and outer photoreceptor changes suggestive of CSC, whereas the foveal OCT scan of the left eye was normal. Extrafoveal OCT scanning in the left eye (*t* and *v*; upper and lower scan in *r*) showed RPE/outer photoreceptor changes without SRF, despite the possible hot spot of leakage on FA. At screening, subfoveal CT was 232 μm in the right eye and 274 μm in the left. On OCT, foveal SRF in the right eye (**u**) had decreased after 6 weeks of follow-up.

**Fig. 2:**
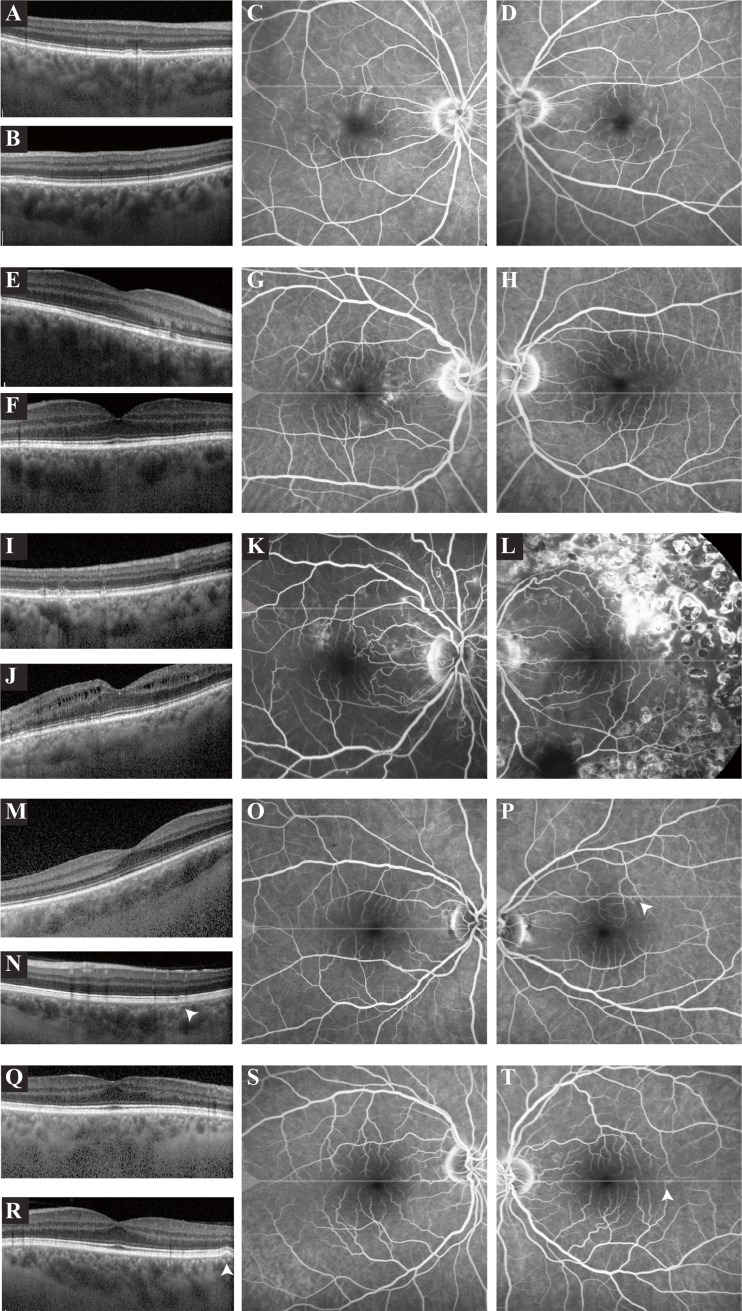
Ophthalmological imaging of five patients with primary hyperaldosteronism in whom retinal pigment epithelium (RPE) changes suggestive of subclinical central serous chorioretinopathy (CSC) were detected at screening. **a–d**. Extrafoveal optical coherence tomography (OCT) scanning of the right (**a**) and the left eye (**b**) of this 72-year-old male patient revealed mild outer retinal changes. Mild diffuse macular hyperfluorescent changes were detected on fluorescein angiography (FA) of the right (**c**) and the left eye (**d**). **e–h**. On the foveal OCT scan of this 50-year-old male patient, subtle outer retinal changes were found in the right eye (**e**), with a normal OCT in the left eye (**f**). FA of the right eye (**g**) showed diffuse hyperfluorescent changes, and a mild hyperfluorescence in the macula of the left eye (**h**). **i–l**. Extrafoveal OCT scan of the right eye (**i**) of a 59-year-old male patient revealed mild hyperreflective focal irregularities on the photoreceptor/RPE level. On the foveal OCT scan of the left eye (**j**) intraretinal edema was observed. FA of the right eye (**k**) of this patient showed multifocal irregular hyperfluorescent changes, with some peripapillary indistinct focal hyperfluorescent areas suggestive of small leaking hot spots, despite an absence of serous SRF on OCT. FA of the left eye (**l**) showed scars of previous scatter laser treatments performed elsewhere for a retinal venous occlusion. **m–p**. OCT scan (**m**) and FA of the right eye (**o**) of this 36-year-old female patient revealed no abnormalities. OCT scanning of the area superior to the macula of the left eye (**n**) revealed mild outer retinal changes, with corresponding mildly hyperfluorescent changes on FA (**p**; *arrowhead* in *n* and *p*). **q-t**. OCT scan (**q**) and FA of the right eye (**r**) of a 58-year-old male patient showed no abnormalities. Foveal OCT scanning of the left eye (**s**) revealed a small RPE detachment, with corresponding mildly hyperfluorescent changes on FA (**t**; *arrowhead* in *s* and *t*)

On OCT, fovea-involving serous SRF was detected in 2 of 13 patients (15 %; a 64-year-old female and a 74-year-old male patient). Imaging of the female patient, to whom spironolactone 50 mg thrice daily was being prescribed since 2008, showed signs of bilateral chronic CSC (Fig. 1a–i, k–l). On FA, hot spots of leakage were detected in the right eye (Fig. 1e). As part of standard clinical care, she underwent indocyanine green angiography, which showed polypoidal choroidal vasculopathy on the border of an area of a possible occult neovascularisation (Fig. 1f). The patient was scheduled for full-dose photodynamic therapy, leading to disappearance of SRF at the evaluation visit 6 weeks later (Fig. 1j). At the follow-up visit 3 months after PDT, no SRF was present. Ophthalmological imaging in the male patient (Fig. 1m–t, v), who was using eplerenone 100 mg twice daily since 2012 and in whom bilateral diffuse hyperfluorescent areas were detected on FA (Fig. 1q-r) showed serous SRF (Fig. 1s). The SRF had decreased at the scheduled visit 6 weeks later (Fig. 1u). OCT imaging revealed outer photoreceptor/RPE changes reminiscent of changes in chronic CSC in 5 of the remaining patients (38 %), which was supported by corresponding hyperfluorescent changes on FA (Fig. 2a–t). Out of these patients, only 1 patient was using spironolactone since 10 weeks. Fundus autofluorescence showed variable mild hypo- and hyperfluorescence of the lesions. The blood pressure of one of these patients was not adequately controlled. In the other hypertensive patient, no ophthalmological pathology could be detected. Ophthalmological phenotyping of the three patients who reported current or previous steroid use did not reveal any abnormalities. In all 13 patients, mean subfoveal CT on EDI-OCT was 290.2 ± 65.0 μm (range, 123–377 μm). Ophthalmological characteristics of the patients are summarized in Table 2.

**Table 2:** Ophthalmological characteristics of patients with primary hyperaldosteronism

Patient	Steroid Use	History of chorioretinal disease	BCVA OD	BCVA OS	SPH EQ OD	OCT OD	SCT OD (μm)	OCT OS	SCT OS (μm)	FAF OD	FAF OS	FA OD	FA OS	Figure
1	–	Choroidal naevus (right eye)	91	91	+1.75	Outer retinal changes	367	Outer retinal changes	356	–	–	Diffuse macular hyperfluorescent changes	Diffuse macular hyperfluorescent changes	2A-2D
2	–	“Early age-related macular degeneration” (right eye)	67	77	−0.25	PCV, foveal SRF, RPE detachments, RPE alterations	235	RPE alterations	206	Multifocal FAF changes	Multifocal FAF changes	Temporal hot spots of leakage, hyperfluorescent spot under the fovea	Hyperfluorescent changes	1A-1L
3	–	Venous hemi-occlusion (left eye)	98	91	+2.00	Mild hyperreflective focalirregularities	284	Intraretinal edema	281	–	–	Diffuse hyperfluorescent changes, with a possible small hot spot of leakage	Scars of previous scatter laser treatments	2I-2L
4	–	–	89	85	−1.00	–	287	–	268	–	–	–	–	
5	Cutaneous (2013)	–	88	91	−6.25	–	123	–	128	–	–	–	–	
6	–	–	95	93	−1.25	–	305	–	302	–	–	–	–	
7	–	–	84	93	+5.00	Outer retinal changes	341	Outer retinal changes	351	Focal FAF changes	–	Diffuse hyperfluorescent changes	Mild macular hyperfluorescent changes	2E-2H
8	–	–	89	90	0	–	357	Outer retinal changes	315	–	–	–	Hyperfluorescent changes	2M-2P
9	–	–	89	86	+0.50	–	377	RPE detachment	372	–	–	–	Hyperfluorescent changes	2Q-2T
10	–	–	86	94	+3.00	Foveal SRF, RPE alterations	232	RPE alterations	274	Peripapillary FAF changes	Peripapillary FAF changes	Hyperfluorescent changes	Hyperfluorescent changes, with a possible hot spot	1M-1V
11	Oral (2012)	–	84	85	−0.25	–	306	–	289	–	–	–	–	
12	Intranasal (current)	–	99	93	+1.00	–	292	–	302	–	–	–	–	
13	–	–	92	93	−0.50	–	306	–	289	–	–	–	–	

*BCVA* = best-corrected visual acuity*, FA* = fluorescein angiography*, FAF* = fundus autofluorescence, *OCT*= optical coherence tomography, *OD* = right eye*, OS* = left eye*, PA =* primary hyperaldosteronism, *PCV =* polypoidal choroidal vasculopathy*, PDT =* photodynamic therapy*, RPE =* retinal pigment epithelium, *SCT =* subfoveal choroidal thickness*, SPH EQ =* spherical equivalent of the manifest refraction, *SRF =* subretinal fluid

## Discussion

In this study, 7 out of 13 PA patients (54 %) had a variable degree of outer retinal and RPE changes on multimodal imaging. Two of these patients (29 %) had serous SRF leakage in the macula, including a patient with CSC-like changes and unilateral polypoidal choroidal vasculopathy (Fig. 1a–l), and a patient with unilateral CSC with subfoveal fluid and bilateral RPE changes resembling those seen in chronic CSC (Fig. 1m–v). Despite the fact that the latter patients used MR antagonists at the time of ophthalmological evaluation, the abnormalities and serous SRF leakage were present. In the five other patients, retinal abnormalities that could be characteristic of (subclinical) CSC were seen. Although we observed RPE abnormalities and no history of SRF in these patients, mean subfoveal CT at the time of evaluation in the current case series of PA patients was comparable with the findings in healthy volunteers [25, 26]. Despite the fact that mean subfoveal CT in CSC patients is usually increased compared to healthy subjects [27–29], several authors have described that this is not the case in all CSC patients [28, 29]. The small number of included patients and the relatively high age of these possible CSC patients could also have contributed to the normal subfoveal CT in this study [30].

Based on the results of this study, aldosterone and the MR-mediated pathway may play a role in the pathogenesis of CSC-like changes in patients with PA. Aldosterone and cortisol are adrenal cortical steroid hormones that exert their actions via the MR and GR. It is unclear how aldosterone and the MR may be linked to the pathogenesis of CSC. The MR is present in the choroid, the RPE, and neurosensory retina, as observed in humans, rats, and monkeys [2, 15, 31–33]. Aldosterone stimulation of the MR may affect the choroidal vascular system through various effects, such as induction of oxidative stress, inflammation, hypertrophic remodeling, fibrosis, and endothelial dysfunction [34, 35]. The two physiological ligands aldosterone and cortisol are able to bind to the MR with similar affinity. In epithelial tissues and the choroid, MR acts as a physiological aldosterone receptor. It is protected from being activated by glucocorticoids by the enzyme 11ß-hydroxysteroid dehydrogenase type 2, which metabolizes cortisol into inactive cortisone [36]. Like the MR, this enzyme is present in the choroid, RPE, and neurosensory retina [31–33]. Without the conversion, MR acts as a cortisol receptor, as blood levels of cortisol are much higher compared to aldosterone levels, even when binding of cortisol to corticosteroid binding globulin is taken into account [37]. The effect of PA suggests either overactivation of aldosterone-preferring MRs or occupancy of cortisol-preferring MRs, for example during periods in the circadian cycle when cortisol is normally very low. In a rat model, MR overexpression increased CT, and resulted in dilation of choroidal vessels, focal disruption of RPE tight junctions, and RPE detachments [2]. Preliminary clinical studies also indicate that eplerenone and spironolactone have a beneficial effect on CSC, although their exact role in the treatment of CSC is somewhat controversial due to possible variable and temporary treatment effects and a lack of randomized placebo-controlled trials [17–20]. Furthermore, there does not seem to be a marked effect of these treatments on CT [19]. Apart from a possible direct role of the MR pathway in the pathogenesis of retinal abnormalities in this PA patient cohort, effects of prolonged hypertension (which was present in all patients) may have also played a role. Moreover, MR antagonists were prescribed to both patients in whom macular SRF was detected at the moment of ophthalmological phenotyping. The relatively severe PA in the patient group that received the ophthalmological phenotyping could also have contributed to the results of this study [24].

In conclusion, findings suggestive of (subclinical) CSC were detected in most patients with PA. Ophthalmologists who treat CSC patients with hypertension of unknown origin should thus have a high index of suspicion for PA, in particular because of the underestimated incidence of PA. In dealing with PA patients, clinicians also need to be aware of the potential coexistence of CSC, which may remain subclinical.
